# What Is Karyotype Coding and Why Is Genomic Topology Important for Cancer and Evolution?

**DOI:** 10.3389/fgene.2019.01082

**Published:** 2019-11-01

**Authors:** Christine J. Ye, Lukas Stilgenbauer, Amanda Moy, Guo Liu, Henry H. Heng

**Affiliations:** ^1^The Division of Hematology/Oncology, Department of Internal Medicine, University of Michigan, Ann Arbor, MI, United States; ^2^Center for Molecular Medicine and Genomics, Wayne State University School of Medicine, Detroit, MI, United States; ^3^Department of Pathology, Wayne State University School of Medicine, Detroit, MI, United States

**Keywords:** chromosomal instability (CIN), fuzzy inheritance, genome chaos, genome theory, karyotype or chromosomal coding, missing heritability, non-clonal chromosome aberrations (NCCAs), system inheritance

## Abstract

While the importance of chromosomal/nuclear variations vs. gene mutations in diseases is becoming more appreciated, less is known about its genomic basis. Traditionally, chromosomes are considered the carriers of genes, and genes define bio-inheritance. In recent years, the gene-centric concept has been challenged by the surprising data of various sequencing projects. The genome system theory has been introduced to offer an alternative framework. One of the key concepts of the genome system theory is karyotype or chromosomal coding: chromosome sets function as gene organizers, and the genomic topologies provide a context for regulating gene expression and function. In other words, the interaction of individual genes, defined by genomic topology, is part of the full informational system. The genes define the “parts inheritance,” while the karyotype and genomic topology (the physical relationship of genes within a three-dimensional nucleus) plus the gene content defines “system inheritance.” In this mini-review, the concept of karyotype or chromosomal coding will be briefly discussed, including: 1) the rationale for searching for new genomic inheritance, 2) chromosomal or karyotype coding (hypothesis, model, and its predictions), and 3) the significance and evidence of chromosomal coding (maintaining and changing the system inheritance-defined bio-systems). This mini-review aims to provide a new conceptual framework for appreciating the genome organization-based information package and its ultimate importance for future genomic and evolutionary studies.

## Introduction

Sequence-driven and gene-focused molecular research has surprisingly revealed its key limitation: the predictive value between content of individual genes and cellular or organismal phenotype is not strong, especially when dealing with many common and complex diseases like cancer (Heng, 2015). This limitation is at odds with many promises that rationalized the need of various large-scale sequencing and -omics projects (van Karnebeek et al., 2018). Moreover, combined with missing heritability (Eichler et al., 2010; Zuk et al., 2012), these limitations fundamentally challenge the gene theory where the inheritance of a group of individual genes is the key causative factor of phenotype (Heng, 2009; McClellan and King, 2010; Boyle et al., 2017), even though this issue is rarely discussed in public.

Since the 10th-anniversary celebration of the completion of the Human Genome Project, different reasons have been offered to explain these limitations (Check Hayden, 2010). The article “Genomics is not enough” (Chakravarti, 2011) called into question how adequate is the concept of the gene and the general genomic mechanism of diseases based on sequencing data.

Unfortunately, when highly heterogeneous data do not fit the expectation of pattern identification, the data are often blamed. One general conclusion is that the current genomic data are either not enough (quantity) or not good enough (quality). Logically, the future research should focus on the data: how to generate and collect more data and how to improve data analyses. Suggested approaches include: 1) collect additional data sets from more clinical samples and develop better computational platforms to filter out the “noise” and to identify the patterns; 2) incorporate epigenetics, gene–environment interactions, microbiota, and metabolic profiles into the analyses; and 3) use the combinatorial approach of systems biology (Ao et al., 2010; Palsson, 2015).

Others are less certain about how to move the field forward (Weinberg 2014). The complexity in genomic medicine requires a new framework to understand the heterogeneous data and its implications. Our group considers biosystems as adaptative systems and focuses on evolutionary mechanisms rather than specific molecular mechanisms. While it is challenging to understand the common mechanism of genomics through reductionist approaches (focusing on genetic parts characterization), it can be achieved by studying the evolutionary mechanism (tracing the pattern of evolution and system emergency). Clearly, studying the genome-mediated somatic evolution will be a better strategy than characterizing gene-based mutations or pathways, as many diverse pathways can lead to the same evolutionary end-products, and each “run” of somatic evolution will likely produce different genomic landscapes.

Recently, cancer genome projects have validated our main predictions about the importance of genome-mediated somatic evolution and limitations of gene-focused research. The increased sample size in most cancer types confirmed the high degree of genomic heterogeneity as a general rule (one which cannot simply be eliminated by bioinformatics tools). The chromosomal profile provides better clinical predictions than gene mutation profiles (Jamal-Hanjani et al., 2017), and the genome chaos, including chromothripsis, can be detected from many cancer types, challenging the stepwise gene mutation theory of cancer (Ye et al., 2018a; Ye et al., 2018b). Furthermore, genome-mediated evolution has received increased attention, as it is linked to system stress, immuno-response, transcriptional dynamics, and cancer evolutionary potential (Horne et al., 2014). Chromosomal changes, including mosaicism, are a universal feature in many common and complex diseases (Iourov et al., 2008a; Iourov et al., 2008b; Iourov et al., 2012; Heng et al., 2016; Iourov et al., 2019). Equally important, the integrity of the karyotype has been linked to the function of sexual reproduction and is the main system constraint of macro-evolution for organisms (Heng, 2007b; Wilkins and Holliday, 2009; Gorelick and Heng, 2011); the genome organization has been considered the organizer of network interaction (Heng, 2009). Such realization has established the core genome or karyotype, rather than individual genes, as the evolutionary selective package.

Altogether, chromosomal-related research is regaining its popularity. As mentioned by editors of this special issue, chromosome biology represents the key to understanding disease mechanisms, genome architecture, and evolution, as genetic inheritance relies on the proper organization of chromosomes and the genome. However, influenced by the gene-centric tradition, recent chromosomal studies are still focusing on gene-defined “parts inheritance.” Rather than address the mechanism of how chromosomes organize the expression and interaction of individual genes, many still consider chromosomes the vehicles or helpers of genes (e.g., contributing to epigenetics by modifying the gene’s function).

Here, a newly realized key concept—order of DNA sequence on chromosome serving as a system code—is briefly discussed. Although “system inheritance” has been previously promoted (Heng, 2009), it has failed to transform the field, possibly due to the dominance of gene-centric concepts and the high hopes for various large-scale sequencing projects. With the recent discussions of karyotype or systematic chromosome-sets-coded inheritance (Heng, 2019), the time is ripe to embrace this new framework, which should serve as a foundation for future genomic research.

## Karyotype Coding Defines System Inheritance

### Rationale and Metaphor for Searching for New Genomic Inheritance

A key rationale of searching for new types of inheritance is because gene-based inheritance has several limitations. First, missing heritability is a real phenomenon rather than a methodological limitation caused by insufficient samples or technologies, which challenges both current technical strategy and the concept of genes. Second, many case studies have illustrated a lack of correlation between gene profile and phenotype, with strong correlation only being detectable from exceptional cases. Third, for cancer research, chromosomal alterations are abundant, most of which differ from gene mutation (except in the cases of gene fusion caused by chromosomal translocation). Furthermore, gene mutation and epigenetic effects do not explain macro-cellular evolution. Fourth, while interesting, most epigenetic regulation involves fine-tuning of gene regulation, which is not sufficient to explain the missing inheritance.

Since multiple levels of genomic organization comprise eukaryotic systems, and a given chromosomal change often can impact many genes, chromosomal alteration represents information change at a higher system level. Equally important, the bio-topological features serve as an important form of bio-information. The specific chromatin distribution within 3D nuclei highlights the topological significance regarding the gene’s relationship along and among chromosomes.

Thus, studying inheritance as defined by chromosome sets should be the priority, especially knowing that this level has been traditionally ignored and deserves timely and systematical study. The topological context of the chromosome set likely serves as the context of gene interaction at the scale of an entire genome.

To illustrate the importance of bio-topology in defining the function of the system, the relationship between building materials (e.g., bricks) and the overall structure of buildings (e.g., architecture) can serve as a metaphor, where the information encoded within architecture can be independent from information encoded within the materials, and the same materials can be used to build different structures with different functions. This metaphor serves to show that sequencing all genes to decode the genomic blueprint will not work. The coding of how genes interact rather than how an individual gene makes protein is the blueprint. The topological relationship among genes likely serves as the genomic information.

### Karyotype Coding: Hypothesis, Model, and Prediction

By considering the genomic topology as a new type of information, we have hypothesized that chromosome sets carry organizational information of genes (system inheritance) that is distinct from the information created strictly by individual gene sequences (parts inheritance). The system inheritance is unique for most species and is maintained by sexual reproduction through meiosis (the main function of chromosomal pairing in meiosis serves as a major checkpoint to maintain the correct order of the chromosomal coding) (Heng, 2007a). Chromosomes are not just the vehicle of genes but the organizers of gene interaction (by providing the physical platform of the genetic network). We have additionally posited that this genomic information can be reshuffled *via* chromosomal rearrangement to create a new emergent genome with new system inheritance.

**Figure 1:**
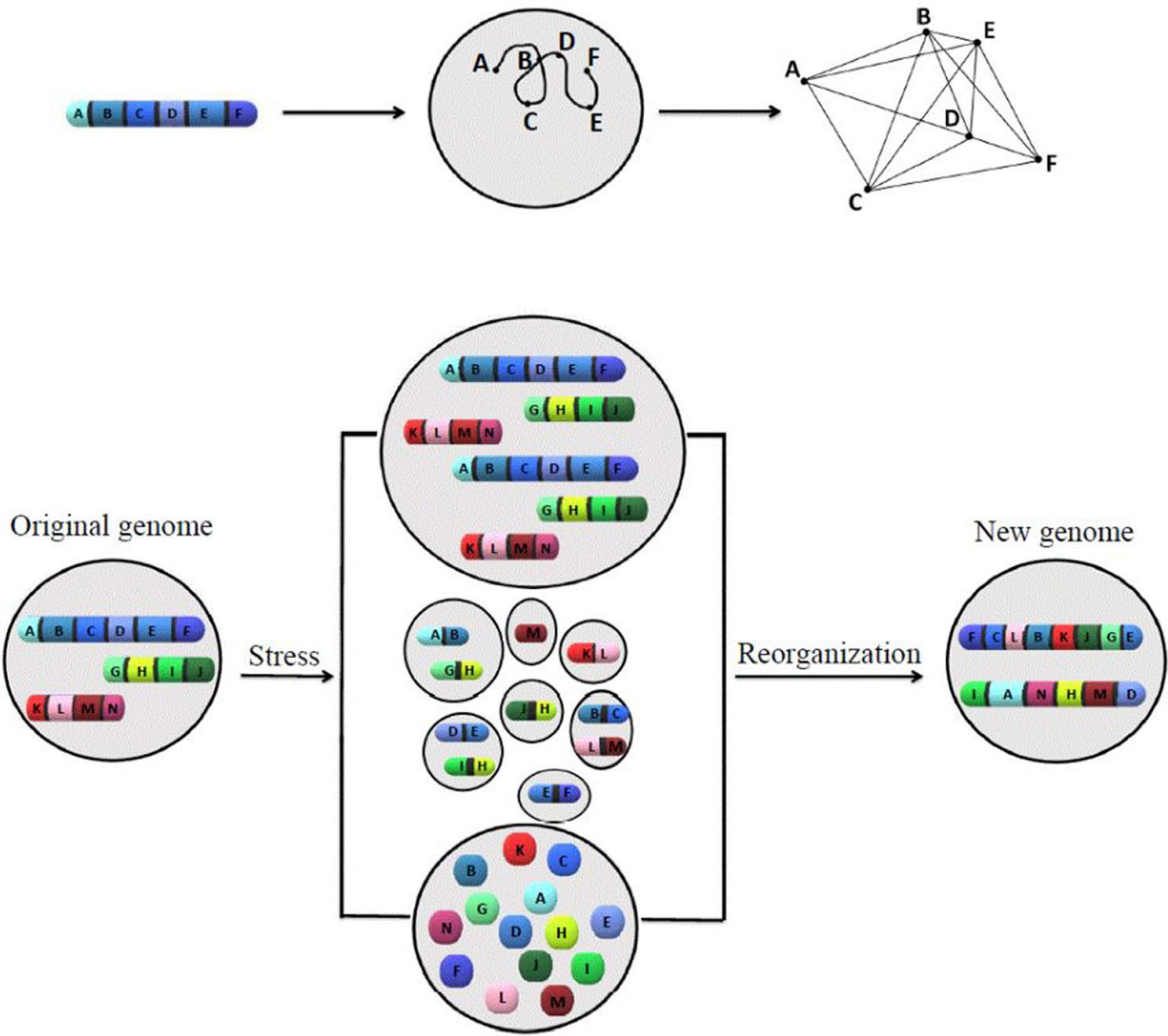
The model of how karyotype or chromosomal coding defines the network structure, and how chromosomal/nuclear variation changes the chromosomal-coded system inheritance. The proposed models to illustrate the relationship between order of genes along chromosomes, network structure (upper panel), and how stress-induced genome re-organization creates a new genome through genome chaos (lower panel). The upper panel illustrates one chromosome with a gene order of A to F, its chromatin domain in interphase nuclei, and a defined network structure (from left to right). For simplicity, only one chromosome is shown. The pattern of interaction among multiple chromosomes would be more complicated. The lower panel illustrates the process of new genome emergence (from the original genome through different types of chromosome/nuclear re-organization under crisis). Only three chromosomes are presented for the original genome. Under high levels of cellular stress, genome chaos occurs as an effective survival strategy. Among many types of genome re-organization (including different types of genome chaos), only polyploidy (upper), micronuclei clusters (middle), and chromosomal fragmentation (lower) are shown. Additional types of genome chaos can be found in Heng et al. (2013a), Liu et al. (2014), and Heng, 2019. The result of genome re-organization (not dependent on the mechanism in which it proceeds) is the formation of new genomes with a higher chance of survival and new chromosomal codes reflected by two newly formed chromosomes with new gene order, providing new network structures.

A model has been introduced (**Figure 1**) to summarize ideas behind karyotype coding. This model illustrates the relationship between the order of genes along chromosomes and its defined network structure, as well as how stress responses change the system coding.

The following are key features, observations, and predictions supporting karyotype-coded system inheritance (see also **Table 1**)

- The majority of cancer cells and natural species display different karyotypes. Different species have their own unique gene order along and among chromosomes.
- Alteration of gene order along the chromosome is biologically significant. The synteny relationship (conservation of gene order) among different species is well known, and the positional effect and the importance of order of genes within a gene cluster is well appreciated (e.g., Hox cluster, topoisomerase associate domains (TADs), and position effect). Recently, the significance of order of genes has been illustrated in synthetic biology (Heng, 2019).
- Chromosomal alterations (e.g., translocations, aneuploidy, and polyploidy) can alter system inheritance as reflected by the transcriptome and the phenotypes. It also can trigger genome instability to produce further chromosomal changes.
- Changing the coding is a common mechanism for new genome formation for both organismal and somatic evolution. Chromosomal re-organization creates new emergent information and is the most effective way of creating new and sometimes drastically different phenotypes.
- A given gene can have different functions within different genomic topology, exhibited through increased or reduced activity, as well as new genomic interaction with other genes, which can change its function. The same protein can have different functions when located in different regions of the cell, with different partners, or when involving different pathways. It is also possible that different cellular sites of protein synthesis are function specific. Nevertheless, most genes are known to work in this context-dependent manner. The genomic topology serves as such context.
- Different karyotypes can have similar phenotypes as long as some functional modules are preserved within an altered genome. Alternatively, different genomes can display different phenotypes in different environments (many new phenotypes only occur in altered “future” environments).
- There is a gene and karyotype interaction (both collaboration and conflict). The genome can control or influence an individual gene’s function. The change of genomic context also includes gene–promoter interaction. For example, the capture of an aerobic promoter by *Escherichia coli* with a previously anaerobic or unexpressed citrate transporter leads to a novel phenotype (van Hofwegen et al., 2016).
- The gene’s key evolutionary involvement is mainly at the micro-evolutionary phase.
- Fuzzy inheritance can be detected from the chromosomal coding level as well. Furthermore, the heterogeneity of the karyotypes can be explained by the “core genome” concept (Heng, 2019).

**Table 1:** Terminology/rationales/evidences/implications of karyotype-coded system inheritance.

I.	Terminology
a.	“Karyotype coding” or “chromosomal (set) coding” functions as an organizer of gene interactions within the entire genome. Its biological effect is not just on individual genes but on the entire genomic network. As opposed to gene coding or vague ideas that chromosomes carry additional information, karyotype coding is defined by specific features: 1) the physical organization of the chromosome codes system information; 2) genomic topology provides context for individual genes; and 3) since different species display unique karyotypes or core genomes, karyotype coding is often species-specific. The key is that the order of gene and non-coding sequences along a chromosome represents a new “system inheritance,” much like how the order of base pairs codes for “parts inheritance” in mainstream “gene coding.”
b.	Although the physical location of individual genes along a chromosome has previously been linked to gene expression (such as the position effect), karyotype coding has long been ignored. However, there have been efforts to search for inheritance above the gene coding level. For example, the “genome system architecture” concept proposed a model based on how a computer program or operating system is organized (Shapiro, 2005). Specifically, the distribution pattern of repetitive DNA was suggested as a key architectural factor. Karyotype coding is described by the order of genes and non-gene genomic sequences, including repetitive sequences and sequences for chromatin architecture such as topoisomerase associate domains (TADs).
**II**.	**Brief rationales/history of search for new inheritance**
a.	Chromosomal position effect has long been observed to impact chromatin behavior and function of genes (Heng et al., 2004; Elgin and Gunter, 2013; Heng, 2019)
b.	Missing heritability is real and a search for inheritance beyond genes is urgently needed (Eichler et al., 2010; Heng et al.,2011; Zuk et al., 2012)
c.	Studying cancer evolution illustrates the distinctive roles of inheritance between gene and genome, and the emergence of new karyotypes is key for cancer evolution (Heng et al., 2006; Heng, 2007a)
d.	A collection of gene sequences does not equal the blueprint. Considering biological systems as multiple levels of interaction/control systems requires a higher level of genomic coding (Heng, 2009; Heng et al., 2011)
e.	Genomic topology likely functions as the coding of the genomic blueprint or gene interaction (Heng, 2019)
f.	The function of sex represents a mechanism of preserving chromosomal coding (Heng et al., 2006; Gorelick and Heng, 2011; Heng, 2015; Heng, 2019)
**III**.	**Evidences to support chromosomal coding**
a.	Each chromosome has its physical domain within a nucleus, and the genomic topology is related to a gene’s function (Cremer and Cremer, 2001; Heng et al., 2004)
b.	The importance of gene clusters in development (Hox genes) (Gehring, 1998)
c.	Chromosomal synteny is preserved among plants and animals (Eckardt, 2001; Murphy et al., 2005)
d.	The formation of new gene clusters contributes to specific pathways (Wong and Wolfe, 2005)
e.	Different karyotypes among species suggest that genomic topology (order of genes/regulation elements) is species specific (Heng 2009)
f.	Chromosomal alterations represent the most common driver for cancer evolution (Erenpreisa et al., 2005; Walen, 2005; Stevens et al., 2007; Heng 2007a; Wallen, 2008; Zhang et al., 2014; Horne et al., 2015; Bloomfield and Duesberg, 2016; Hamann et al., 2017; Hamann et al., 2017; Bakhoum et al., 2018; Salmina et al., 2019)
g.	Changing the chromosomal number by fusing them into one single yeast chromosome can effectively establish reproduction barriers (Luo et al., 2018; Shao et al., 2018)
h.	Chromosomal alterations can rescue yeast following key gene knockout (Rancati et al., 2008)
i.	Individual chromosomal alterations can impact the entire transcriptome (Stevens et al., 2013; Heng, 2015)
j.	The linkage between genome alterations and various diseases is common (Heng 2009; Heng et al., 2016; Heng 2019), and chromosomal mosaicism is a common phenomenon (Iourov et al., 2012; Heng et al., 2013a; Iourov et al., 2008a; Iourov et al., 2008b; Iourov et al., 2019)
k.	Both TADs and position effects are examples of data where the expression of coding information is sensitive to physical location in the genome.
l.	NCCAs, an index of genome instability, have been linked to “fuzzy inheritance,” which is essential for evolutionary potential (Heng 2009; Heng 2019). NCCAs contribute to the emergence of new genomes under crisis, including outlier-mediated drug resistance, various types of cellular survival, and adaptations (Rangel et al., 2017; Poot, 2017a; Frias et al., 2019). Interestingly, other types of genome dynamics, such as small supernumerary marker chromosomes and transposable elements, can influence chromosomal coding under certain conditions (Liehr et al., 2013; Liehr 2016; Poot, 2017a; Poot, 2017b). Equally important, the pattern and dynamics of NCCAs should be used to study somatic mosaicism (Lourov et al., 2008a; Lourov et al., 2008b; Lourov et al., 2012; Biesecker and Spinner, 2013; Heng et al., 2013a; Lourov et al., 2019) and multiple levels of core genome-associated genomic interactions (Heng et al., 2013a; Heng et al., 2016; Shapiro, 2017; Shapiro, 2019; Heng 2019), including minimal genome variations at germline, somatic alteration and mosaicism, and host microbiome. Such genome–environment interactions play an important role for evolutionary adaptation and survival.
**III**.	**Implications**
a.	Reconcile “parts inheritance” and “system inheritance” and prioritize the importance of the true blueprint for eukaryotic systems
b.	Emphasize the importance of using chromosomal dynamics to study cellular evolution, and applying chromosomal aberrations (rather than individual gene mutation profiles) as a biomarker
c.	Understanding the genomic basis of information inheritance in macro- and micro-evolution
d.	Illustrate the emergence of phenotype based on genomic mosaicism and its interactions with all involved genomes and the environment

More examples can be found in Heng (2009, 2019).

### Significance and Evidence: Maintaining and Changing System Inheritance-Defined Bio-Systems

Significance:

- Inheritance is a key feature for all biosystems. Establishing the correct mechanism for how biosystems create, and then pass on, their information is of utmost importance for both basic genomic research and its application for medicine. It is long accepted that the gene defines bio-inheritance. Now with the realization that chromosome-mediated system inheritance organizes the parts inheritance, many bio-concepts based on the understanding of parts inheritance need to be modified, including genomic/evolution studies and molecular medicine.
- The concept of karyotype coding effectively addresses the issue of missing heritability. This key genome factor likely accounts for a large portion of the missing heritability, even though the fuzzy inheritance at gene level is also contributing to the phenomenon. In addition, system inheritance also defines the boundary of the epigenetic regulation; equally important, there is a gap between germline-defined inheritance and the environmental-influenced somatic inheritance (such emergent properties are highly dynamic and constantly changing in response to development, aging, and cellular stress).
- Karyotype coding unifies organismal evolution and somatic evolution, as both evolutions need to pass system inheritance and involve macro- and microevolution. They share the same two phases of macro- and microevolution despite the different mechanisms used to maintain system inheritance. It also explains why cancer can happen within 20–30 years while organismal evolution takes much longer (though initial speciation can be quick, it often takes a long time to form a stable population). Without the genome constraint ensured by sexual reproduction, the genome chaos can fast become dominant in somatic evolution, leading to cancer (Heng, 2015). In contrast, the function of sex provides the strong genome constraint in organismal evolution. For a successful speciation, it requires three highly rare events: genome re-organization to produce survivable individuals with altered genome; the availability of other mating partners with a matching genome (producing fertile offspring); and the initial small population growing into a visible population (Heng, 2019).
- The model (**Figure 1**) unifies diverse molecular mechanisms of genome variations. Although different molecular mechanisms can be linked to each type of chromosomal/nuclear abnormality, they can all be unified under the evolutionary mechanism of re-organizing chromosomal coding. For example, from aneuploidy and/or simple translocation to chaotic genomes, including chromosome fragmentations, micronuclei cluster, polyploidy, entosis, and budding/bursting/fusion, they all can be explained by changes to the genomic information (Walen, 2005; Stevens et al., 2007; Stevens et al., 2011; Zhang et al., 2014; Ye et al., 2019). Evolutionary selection acts on new emergent genomes with new phenotypes and “cares” less which molecular mechanisms are responsible.

Evidence:

Examples of supporting evidences are listed in **Table 1**. More examples can be found in the book *Genome Chaos* (Heng, 2019).

## Future Direction

In 2011, the journal *Cell* asked a few leading genomic researchers “what’s been most surprising” for the human genome? The answers were: “let’s remember the chromosomes”; “variation and complexity”; “a hidden ecosystem”; and “huge heterogeneity.” Interestingly, all issues are directly related to the chromosomal coding-defined system inheritance (Leading Edge, 2011).

Recently, the importance of chromosomal research has become more obvious. For example, chromosomal abnormalities are copious in cancer including various types of genome chaos, and predicting clinical outcomes based on chromosomal data is much better than based on DNA sequencing data (Davoli et al., 2017;Jamal-Hanjani et al., 2017). In addition, chromosomal and nuclear aberrations have been linked to immune response (Mackenzie et al., 2017;Santaguida et al., 2017). The stochastic chromosomal changes, such as non-clonal chromosome aberrations (NCCAs), are used to measure chromosomal instability (CIN) and to explain treatment outcomes (Heng et al., 2006; Heng et al., 2013a; Heng et al., 2013b). Now, it is increasingly clear why high levels of NCCAs should not be ignored, as they reflect the system instability. Furthermore, the evolutionary meaning of altering the chromosomal coding is also applied to the study of other disease types, and organismal evolutionary studies (Heng, 2009; Heng, 2019).

With the introduction of the chromosomal-coding concept, the following tasks need to be achieved to maturate this concept:

- Further illustrate the molecular details of karyotype coding:
  As illustrated in **Figure 1**, the model of genome-topology based inheritance does not offer molecular details of how karyotype coding works. We know that altering the order of genes along a chromosome can change species and/or phenotypes (like how changing the order of the Hox gene cluster leads to abnormal development and chaotic genome changes in the overall transcriptome); however, little is known about which specific mechanisms are involved at whole genome scale. Unlike how DNA codes for proteins, where there is a direct correlation between a three-nucleotide codon in a nucleic acid sequence and a single amino acid in a protein (which is with high certainty), chromosomal coding is more like “gene regulatory codes,” which determine when, where, and what amount of specific proteins are to be produced (which involve diverse mechanisms and less predictability). Further, chromosomal coding may involve more complicated mechanisms due to the large-scale organization, which likely involves emergent behavior. Nevertheless, studies are needed to link the order changes among genes on chromosomes (including translocation, aneuploidy) to interphase changes (dynamics and/or behavior) and specific pathway changes. Moreover, the altered evolutionary potential needs to be studied with these changes. These studies will likely help people accept the concept of chromosomal coding, even though, similar to mechanisms of “gene regulatory codes” (such as control of chromatin packaging), these mechanisms could be less specific when compared to “gene-protein codes”.
- Illustrate the relationship among different types of bio-inheritance:
  To illustrate the significance of karyotype coding, quantitative and comparative studies are needed to rank the contribution of different types of inheritance under different bioprocesses and environments. The following solutions are needed when systematically comparing different types of bio-inheritance: separating germline and somatic cells (germline with the highest constraint, the somatic cell with highest dynamic changes) to compare the germline profile with tissue-specific somatic cell profiles; separating profiles of individual cells and cellular populations; separating the two phases of cancer evolution (cancer formation by creating new genome systems; microevolution to increase the number of cancer systems, by stochastically capturing the oncogenes) (Ye et al., 2018a; Ye et al., 2018b); separating average populations and outliers; and separating normal physiological conditions and pathological conditions.
- Study mechanisms of organismal macro-evolution and how changes in karyotype coding can create new species:
  While the model of how karyotype change leads to speciation has been proposed (Heng, 2007b; Heng, 2019), it has a long way to go before the research community accepts it. Many questions need to be addressed, for example: How universal is chromosomal coding to define species knowing that it is rather common in angiosperms and in animals (Murphy et al., 2005; Heng, 2009; Dodsworth et al., 2016)? How are we to define species without typical chromosomal coding? Answering these questions requires an understanding of how genome-based information is packaged and regulated. The following approaches are useful: 1) creation of a testable model for the chromosomal code, 2) mechanistic study of chromosomal reshuffling to create new emergent information in evolution, and 3) development of working models where the new emergent genomic topology (with the same gene materials) drives a phenotype. In fact, the suggested chromosome shuffling experiments were already partly performed in yeast (see **Table 1**).
- Clinical implications
  Studying karyotype coding has clinical significance. Besides cancer prediction, it can potentially be used in many common and complex diseases. For example, chromosome instability has been proposed as a new general feature for diseases caused by cellular adaptation and its trade-off (see Horne et al., 2014; Heng et al, 2016). Somatic mosaicism needs to be considered as well as it can alter the phenotypes. Equally important, the combination of system inheritance and the fuzzy inheritance will provide a deep understanding of how environmental interaction contributes to disease phenotype based on the genome–environment interaction.

## Author Contributions

CY, AM, and HH, drafted the manuscript. LS and GL participated in the discussion, literature search, and editing of the manuscript.

## Conflict of Interest

The authors declare that the research was conducted in the absence of any commercial or financial relationships that could be construed as a potential conflict of interest.
